# TREM-2**-**p38 MAPK signaling regulates neuroinflammation during chronic cerebral hypoperfusion combined with diabetes mellitus

**DOI:** 10.1186/s12974-019-1688-9

**Published:** 2020-01-03

**Authors:** Jiawei Zhang, Yu Liu, Yaling Zheng, Yan Luo, Yu Du, Yao Zhao, Jian Guan, Xiaojie Zhang, Jianliang Fu

**Affiliations:** 1 Department of Neurology, Shanghai Jiao Tong University Affiliated Sixth People’s Hospital, 600 Yishan Road, Shanghai, 200233 China; 2 Department of Medicine, Shanghai Eighth People’s Hospital, Shanghai, 200235 People’s Republic of China; 3 Shanghai Key Laboratory of Sleep Disordered Breathing, Shanghai Jiao Tong University Affiliated Sixth People’s Hospital, Shanghai, China

**Keywords:** TREM2, p38 MAPK, Chronic cerebral hypoperfusion, Diabetes, Microglia, Neuroinflammation

## Abstract

**Background:**

Diabetes mellitus (DM) and chronic cerebral hypoperfusion(CCH)are both risk factors for cognitive impairment. However, whether DM and CCH can synergistically promote cognitive impairment and the related pathological mechanisms remain unknown.

**Methods:**

To investigate the effect of DM and CCH on cognitive function, rats fed with high-fat diet (HFD) and injected with low-dose streptozotocin (STZ) followed by bilateral common carotid artery occlusion (BCCAO) were induced to mimic DM and CCH in vivo and mouse BV2 microglial cells were exposed to hypoxia and/or high glucose to mimic CCH complicated with DM pathologies in vitro. To further explore the underlying mechanism, TREM-2-specific small interfering RNA and TREM-2 overexpression lentivirus were used to knock out and overexpress TREM-2, respectively.

**Results:**

Cognitive deficits, neuronal cell death, neuroinflammation with microglial activation, and TREM-2-MAPK signaling were enhanced when DM was superimposed on CCH both in vivo and in vitro. Manipulating TREM-2 expression levels markedly regulated the p38 MAPK signaling and the inflammatory response in vitro. TREM-2 knockout intensified while TREM-2 overexpression suppressed the p38 MAPK signaling and subsequent pro-inflammatory mediator production under high glucose and hypoxia condition.

**Conclusions:**

These results suggest that TREM-2 negatively regulates p38 MAPK-mediated inflammatory response when DM was synergistically superimposed on CCH and highlight the importance of TREM-2 as a potential target of immune regulation in DM and CCH.

## Introduction

As the second most common form of dementia, vascular dementia (VaD) is mainly caused by chronic cerebral hypoperfusion (CCH), which is an aging-related process characterized by a persistent reduction of cerebral blood flow (CBF) [1, 2]. Multiple vascular comorbidities, such as obesity and diabetes, can accelerate the reduction of CBF and facilitate the onset and progression of cognitive impairment [3, 4]. DM is a prevalent devastating chronic metabolic disease affecting a number of people worldwide and CCH-induced brain injury is prevalent in diabetic subjects. Diabetic individuals and rodents often exhibit an increasing risk of developing dementia compared with the control group [5].

Recently, a number of mechanisms thought to underlie cognitive dysfunction caused by CCH, including white matter impairment, oxidative stress and neuroinflammation, among which neuroinflammation plays a primary role in the pathophysiology of VaD [6, 7]. Neuroinflammation is mainly manifested by microglial activation and subsequent release of inflammatory factors, ultimately leading to brain tissue damage. In a rodent model of VaD, inflammatory processes were found evidently in the hippocampus, a crucial brain region involved in learning and memor y[8]. A recent study found that activation of microglia induced by CCH promotes the release of pro-inflammatory factors, which further lead to the long-term potentiation (LTP) impairment and cognitive dysfunctio n[9]. In addition, microglial activation and inflammatory response are also involved in individuals and rodents with D M[10, 11]. However, whether hyperglycemia interacts synergistically with CCH to deteriorate the performances on cognitive function and whether it is through the mechanism of neuroinflammation remain unclear.

A number of signaling molecules, including mitogen-activated protein kinases (MAPKs) and triggering receptor expressed on myeloid cells 2 (TREM-2), are involved in the modulation of microglial activation and inflammatory responses [12, 13]. Extracellular signal-regulated kinases 1 and 2 (ERK1/2), p38 MAPK, and c-Jun N-terminal kinases (JNK) are three major MAPKs, which regulate a wide variety of cellular processes and play an important role in regulating the expression of pro-inflammatory cytokines such as tumor necrosis factor α (TNF-α) and interleukin-1β (IL-1β) [14]. TREM-2 is an important innate immune receptor uniquely expressed on the microglia and is involved in down-regulating neuroinflammation in the central nervous system (CNS) [15]. Recently, a study confirmed that TREM-2 inhibits TNF-α induced inflammatory responses in fibroblast-like synoviocytes via inhibiting p38 pathway activation [16]. Nevertheless, it is unknown how TREM-2 interacts with MAPKs and participates in the regulation of neuroinflammation.

To illustrate whether DM exacerbates the pathologies of CCH and whether neuroinflammation are involved in the process, rats treated with HFD-STZ and BCCAO and mouse BV2 microglial cells exposed to hypoxia and high glucose were used to mimic the pathologies of CCH complicated with DM in vivo and in vitro, respectively.

## Methods

### Animals

Male Sprague-Dawley (SD) rats (160–180 g, 6 weeks old) were housed in cages under controlled temperature (20–25 °C) and light (12 h light/12 h dark) conditions, with water and food available ad libitum. All procedures were performed in accordance with the guidelines of the Medical Experimental Animal Administrative Committee of Shanghai and in accordance with the principles outlined in the National Institutes of Health (NIH) Guide for the Care and Use of Laboratory Animals.

### Model of diabetic rats with chronic cerebral hypoperfusion

**Fig. 1:**
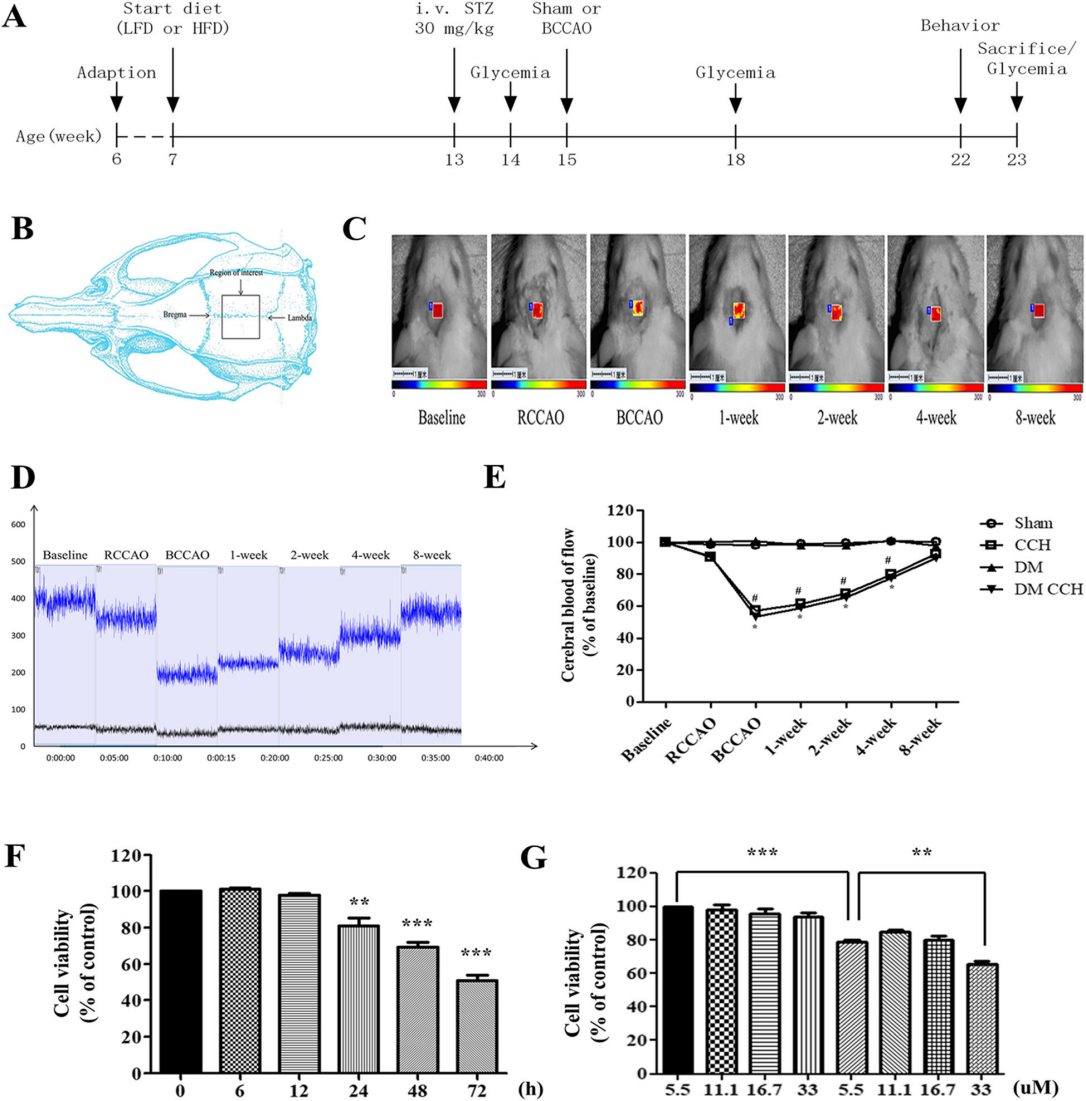
Models of DM and CCH were established in vivo and in vitro. **a** Schematic representation of the experimental protocol. All experiments were performed on the indicated weeks. (B-E) Detection of cerebral blood flow changes by LSCI. **b** The field of view (also known as the ROI) was 5 × 5 mm. **c** Representative contrast and photographic images of cerebral perfusion. **d** CBF absolute values at the following time points: baseline, immediately before BCCAO, immediately after BCCAO, and at 1, 2, 4, 8 weeks after BCCAO. *Baseline* refers to cerebral perfusion before occlusion. **e** Quantification of relative CBF in the ROIs (% of baseline for each animal). Values are expressed as the mean ± SEM (*n* = 3). *P < 0.05, CCH vs. Sham; ^#^*P* < 0.05, DM CCH vs. DM. **f**, **g** Cell viability was determined using CCK-8 assay. Cell viability was decreased under hypoxia and hyperglycemia condition. **f** Cells were exposed to hypoxia (3%) for 24 h under normal glucose condition (5.5 mM). **g** Cells were exposed to different concentrations of glucose (5.5, 11.1, 16.7, or 33 mM) for 24 h prior to a 24 h normoxic (21% O_2_) or anaerobic (3% O_2_) incubation. Data are presented as the mean ± SEM from three independent experiments performed in triplicate. **P < 0.01; ***P < 0.001 compared with the control group

After 1 week of adaptive feeding, the rats were randomly divided into four groups: sham non-diabetic rats (low-fat control diet, LFD + sham) (Sham), sham T2DM rats (high-fat diet + streptozotocin, HFD + STZ + sham) (DM), hypoperfused group (LFD + BCCAO) (CCH), and hypoperfused T2DM rats (HFD+STZ+BCCAO) (DM CCH) (each group *n* = 9). The T2DM rat model was developed according to the method in our previous study with slight modifications [17]. LFD consisted of 10% fat, 70% carbohydrate, and 20% protein, while HFD consisted of 60% fat, 20% carbohydrate, and 20% protein. Both the LFD and HFD were purchased from Research Diets Inc. (USA). Six weeks after the start of LFD or HFD feeding, animals fed with HFD were injected intraperitoneally with a low dose of STZ (30 mg/kg) that was prepared in pH 4.5 citrate buffer while LFD rats only received an equivalent volume of citrate buffer. One week after the STZ injection, 1.0 mL of blood each was collected from the caudal vein of the rats. Rats were recognized as diabetic rats when glucose levels reached 16.7 mM after 1 week of STZ injection. Two weeks after STZ injection, animals were subjected to either sham treatment or two-step BCCAO according to the procedure previously described [18]. Briefly, rats were anesthetized intraperitoneally with chloral hydrate (350 mg/kg). A midline incision was performed to expose both common carotid arteries and gently separated from the vagus nerve. In the CCH and DM CCH group rats, the common carotids were double-ligated tightly using silk sutures 4–0 1 week apart, with the right common carotid artery being occluded first. The sham and DM group rats received the same procedure without carotid artery ligation. During the surgery, a heating pad was used to continuously monitored and maintained the rectal temperature at 37 ± 0.5 °C. Among the 18 rats in the BCCAO groups, two (11%) died one day after surgery, which may be caused by excessive anesthesia or surgical injury. The details of study design are presented in Fig. 1a.

### CBF monitoring by laser speckle contrast imaging

Ten additional rats were used to monitor cerebral blood flow (CBF) changes via laser speckle contrast imaging (LSCI), a technique based on speckle contrast analysis that provides an index of blood flow [19]. Before removing the skin and tissue surrounding the skull, each rat was anesthetized and placed into a stereotactic frame. Using a dental drill, thin a 5 × 5 mm^2^ cranial window of the cerebral cortex (5 mm away from the sagittal suture, between the bregma and the lambdoid suture) to transparency being careful to flush the surface with cold saline frequently in order to avoid damaging the brain. In this study, changes in CBF before and after occlusion was measured using a perfusion speckle imager (Perimed, Stockholm, Sweden). A laser non-contact probe was positioned approximately 10 cm above the frontal-parietal cortical region of the brain.

### Morris water maze test

Morris water maze (MWM test) was performed after 7 weeks of the BCCAO surgery to assess for spatial learning and memory abilities. The utilized maze was a round tank (height 50 cm, diameter 180 cm), divided into four quadrants, filled up to a depth of 30 cm with tepid water (25 ± 1 °C). The water was made opaque by the addition of charcoal-black ink. A movable square hidden platform, 10 cm in diameter, was submerged about 2 cm below the water surface at the 2nd quadrant of the water maze. The maze was surrounded by white curtains with visual cues of four different shapes and sizes placed in the four quadrants. Hidden platform task consisted of four trials per day on five consecutive days. In every trial, rats were gently placed in the water from different quadrants facing toward the inside wall of the tank. In the maze, rats were allowed to swim for a maximum time of 60 s. Once a rat located the platform, it was permitted to remain on it for 20 s. If a rat failed to locate the hidden platform within 60 s, then they were gently guided to the platform and were demanded to remain on the platform for 20 s. During each trial, the latency required to reach the platform was measured as the escape latency. The platform was withdrawn at the sixth day of training for probe trial. The rats were released from the 4th quadrant which is opposite to the target quadrant and allowed to navigate freely for 60 s. During the probe trial, the time spent in the quadrant where the platform had been placed, the number of times across the retracted platform, and the average swimming speed was recorded.

### Immunostaining

Immediately after the Morris water maze test, rats were transcardially perfused with 0.9% saline followed by 4% paraformaldehyde. After the process of perfusion, the brains were collected and fixed in 4% paraformaldehyde for 24 h. Subsequently, the brains were separated into two hemispheres, one of which was embedded in paraffin and sectioned into 5-μm thick slices by a microtome for hematoxylin and eosin (HE) staining, while the other was immersed in a tube containing optimal cutting temperature compound (OCT) for 1 day at − 20 °C and then stored at − 80 °C until they were processed.

For histological analysis, brain tissue sections were deparaffinized, rehydrated, stained with HE according to the manufacturer’s instructions. To performed Immunofluorescent staining, sections were processed for immunofluorescent labeling with ionized calcium binding adapter molecule 1 (Iba1) antibody (for microglia activation) and NeuN antibody (for neurons). First, sections were immersed in blocking buffer (3% donkey serum and 0.3% Triton X-100 in PBS) at room temperature for 1 h, and then incubated overnight at 4 °C with either NeuN antibody (rabbit, 1:500, Abcam), or Iba1 antibody (goat, 1:500, Abcam). After three times of rinsing with washing buffer, either donkey anti-rabbit secondary antibody (Alexa Fluor 488®, 1:500, CST) or donkey anti-goat secondary antibody (Alexa Fluor 594®, 1:500, CST) was incubated for 1 h at room temperature. After three times of washing in PBS, sections labeled with Iba1 antibody were counterstained with 4′,6-diamidino-2-phenylindole dihydrochloride (DAPI, for the cell nucleus) followed by covering with anti-quenching fluorescence mounting medium. The sections were visualized under an Olympus microscope. Digital images were captured from hippocampus at 5 × 10 magnification, from the CA1 and CA3 region and cortex at 40 × 10 magnification, and from dentate gyrus (DG) at 20 × 10 magnification. NeuN-positive cells were quantified in three coronal sections at 100-um intervals in each rat.

### Cell culture

Immortalized mouse BV2 microglial cell lines were purchased from ScienCell (CA, USA) and cultured in MEM medium with 10% FBS and 1% penicillin/streptomycin. For high-glucose treatment, d-glucose was added into a regular medium to make in vitro concentrations of 11.1, 16.7, and 33 mM, which are similar to in vivo levels of glucose under “diabetes mellitus,” “diabetic ketoacidosis,” and “hyperglycemia hyperosmolar status” conditions, respectivel y[20]. For normoxia culture, cells were maintained at 37 °C in a humidified atmosphere containing 95% air and 5% CO_2_. Based on our pilot studies, a higher concentration of oxygen (5%) had little effect on cell viability while a lower concentration of oxygen (1%) plus high glucose induced significant cell death in a short time. The oxygen concentration was therefore kept at 3% for hypoxia culture [21]. If without any specific indication, cells were cultured under normoxic conditions for 24 h in medium supplied with high glucose (33 mM) followed by hypoxic conditions (3% O_2_) for 24 h before they were used in the following experiments.

### Cell viability assay

Cell viability was assessed with the CCK-8 assay using 96-well culture plates. After reaching 60% confluence, the cells were exposed to the experimental media with various concentrations of glucose (5.5, 11.1, 16.7, or 33 mM) for 24 h and then left in anoxic conditions for an appropriate duration to induce hypoxia and high glucose condition. After hypoxia and high glucose exposure, CCK-8 solution (10 μL) was added to each well for 2 h at 37 °C and the absorbance at 450 nm was read on a microplate reader (Thermo Scientific). Each experimental condition was repeated in triplicate. In each experiment, at least three parallel wells were set up.

### Transfection

BV2 cells were cultured in six-well plates overnight and transfected with 50 nM TREM-2-specific siRNA or control siRNA(Zorin, Shanghai) by Lipofectamine 3000 reagent (Invitrogen), according to the manufacturer’s instructions. Briefly, TREM-2-targeted siRNA (100 pmol) was diluted in 250 μL of reduced-serum medium Opti-MEM® I Medium (Gibco), mixed with an equal volume of Lipofectamine 3000 Reagent (6 μL) and incubated for 20 min at room temperature. Then, the siRNA/Lipofectamine solution was added directly to the cells and incubated for 48 h. The relative levels of TREM-2 expression were determined by quantitative RT-PCR and Western blot. Following transfection, the cells were exposed to hypoxia and high glucose as described above.

The sequences of siRNAs were

control siRNA: forward ′5-UUCUCCGAACGUGUCACGUTT-3′,

reverse ′5-ACGUGACACGUUCGGAGAATT-3′;

TREM-2-siRNA1: forward ′5-GAUGCUGGAGAUCUCUGGGTT-3′,

reverse 5′-CCCAGAGAUCUCCAGCAUCTT-3′;

TREM-2-siRNA2: forward 5′-GGAGGUACGUGAGAGAAUUTT-3′,

reverse 5′-AAUUCUCUCACGUACCUCCTT-3′;

TREM-2-siRNA3: forward 5′-CCUUGCUGGAACCGUCACCAUT-3′

reverse 5′-UGGUGACGGUUCCAGCAAGGUT-3′

Lentiviral vectors encoding the mouse TREM-2 gene (NCBI ID: NM_031254.2) and a control lentiviral vector were prepared following the manufacturer’s manual (Zorin, Shanghai). Briefly, the lentiviral vectors were purified and then co-transfected with packaging vectors (Zorin, Shanghai) into 293 T cells. The supernatant was collected and concentrated after 48 h. To generate TREM-2 overexpression, BV2 cells were plated, infected with lentiviral particles and 5 μg/ml polybrene (Zorin, Shanghai) and drug selected with puromycin or GFP. The efficiency of transfection was determined by qRT-PCR and Western blot analysis 72 h later.

### qRT-PCR

Total RNA in cultured cells and brain tissues was extracted using the RNAeasy™ animal RNA isolation kit with a spin column according to the manufacturer’s protocol. Isolated RNA was reverse-transcribed into cDNA using the PrimeScript™ RT Master Mix (Perfect Real Time) following the standard protocol. The qPCR assay was conducted using TB Green™ Premix Ex Taq™ II (Tli RNaseH Plus) with the Applied Biosystems 7500 Real-Time PCR System (Applied Biosystems, United States). The amplification parameters were 95 °C for 30 s, followed by 40 cycles of 95 °C for 5 s and 60 °C for 34 s, 95 °C for 15 s, 60 °C for 60 s, and 95 °C for 15 s. Each sample was analyzed in triplicate, and the relative expression of mRNA was calculated after normalization to β-actin. The relative mRNA expression level in the control group (target mRNA/β-actin value) was set to 100%, and the mRNA values in other groups were converted to fold changes after comparison with the control group. All primer sequences used are listed in Table 1.

**Table 1:**
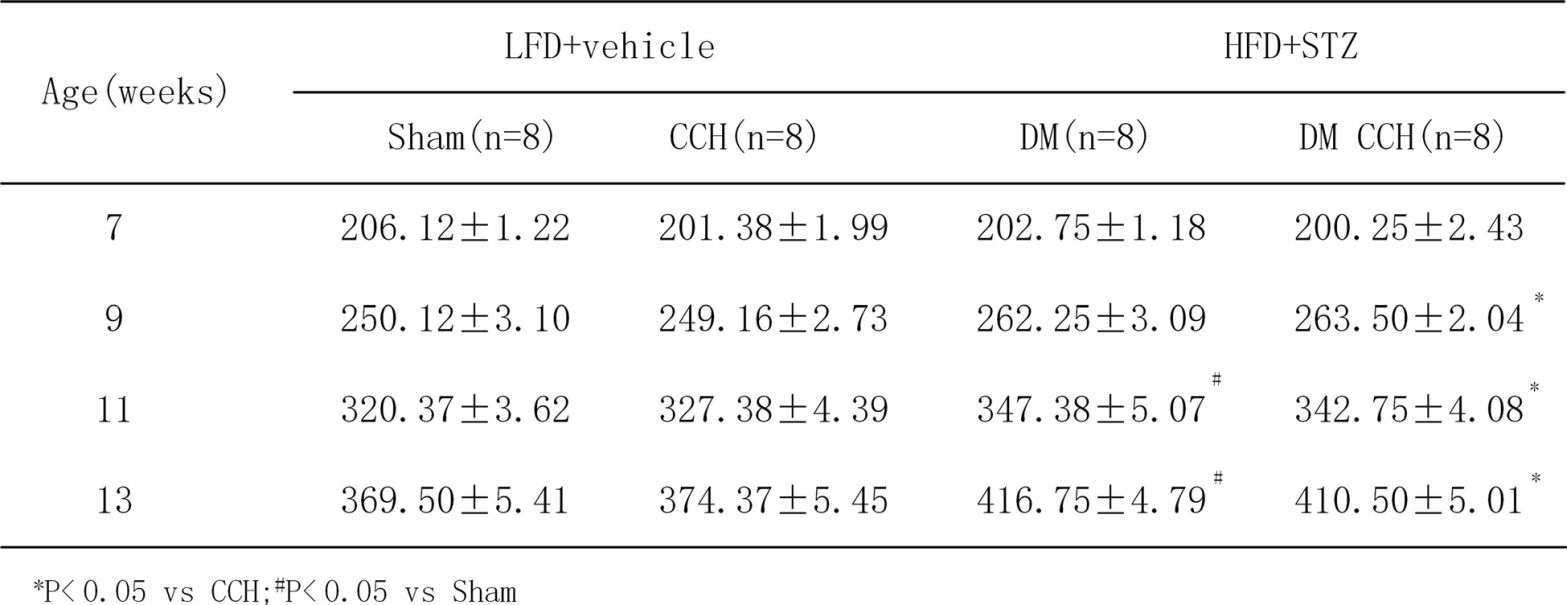
Primers for qPCR

*m* mouse, *r* rat

### Western blot analysis

Cultured cells and brain tissues were lysed with RIPA buffer supplemented with protease and phosphatase inhibitors and collected for protein extraction. The protein concentration was determined using a BCA kit. Different samples with an equal amount of protein were separated by sodium dodecyl sulfate-polyacrylamide gel electrophoresis (SDS-PAGE) and transferred to polyvinylidene fluoride (PVDF) membranes. After blocking with 5% non-fat milk or BSA in TBS-T, the membrane was incubated overnight with primary antibodies (TREM-2, 1:500; p38, 1:1000; p-p38, 1:1000; ERK, 1:1000; p-ERK, 1:1000; JNK, 1:500; p-JNK, 1:500; β-actin, 1:1000) in TBS-T at 4 °C. On the next day, the membrane was washed and then incubated with horseradish peroxidase (HRP)-conjugated immunoglobulin G (IgG) secondary antibodies diluted in TBS-T (1:3000). The protein bands were detected with enhanced chemiluminescence (ECL). Digital images were analyzed using Quantity One to obtain the grayscale value of signals.

### Statistical analysis

Data are reported as mean ± standard error of the mean (SEM) from three independent experiments, each performed in triplicate. The differences among groups were performed using one-way analysis of variance (ANOVA) followed by Tukey’s post hoc test. For the hidden-platform training of the Morris water maze test, the path length was analyzed by two-way repeated-measures ANOVA followed by Tukey’s post hoc test. *P* < 0.05 was considered statistically significant.

## Results

### Stable models of DM and CCH were successfully established in vivo and in vitro

In order to better mimic the clinical characteristics of diabetic patients who suffered from chronic cerebral hypoperfusion, a rat model of DM and CCH was developed in vivo. Firstly, rats were fed chronically with HFD for 6 weeks to induce obesity. The weight of all rats at the start of the study was statistically the same. Table 2 illustrates that the feeding of HFD for 6 weeks resulted in significant increase in body weight in rats as compared with LFD rats. After 6 weeks of dietary manipulation, a single dose of STZ was injected to induce diabetic rats. Compared with LFD rats, HFD-STZ rats had significantly higher blood glucose levels(≥ 16.7 mM) and gradually increased over the study period (Table 3). Two-step BCCAO was considered successful as long as the CBF decreased by ≥ 30% from baseline immediately after surgery. Therefore, LSCI was used to investigate the changes in CBF before and after occlusion. Quantification of relative CBF in the regions of interest showed that BCCAO induced a significant decrease in CBF (57.27 ± 6.35% of baseline, *P* < 0.05) immediately after surgery and then gradually recovered and nearly reached its baseline at 8 weeks after surgery (Fig. 1b–e). These results demonstrate that rats treated with HFD-STZ and BCCAO showed clinical characteristics of type 2 diabetes (T2DM) and CCH. Thus, a stable model of DM and CCH were successfully established in vivo to investigate the effects of diabetes on CCH-induced cognitive deficits and damage in rats.

**Table 2:**
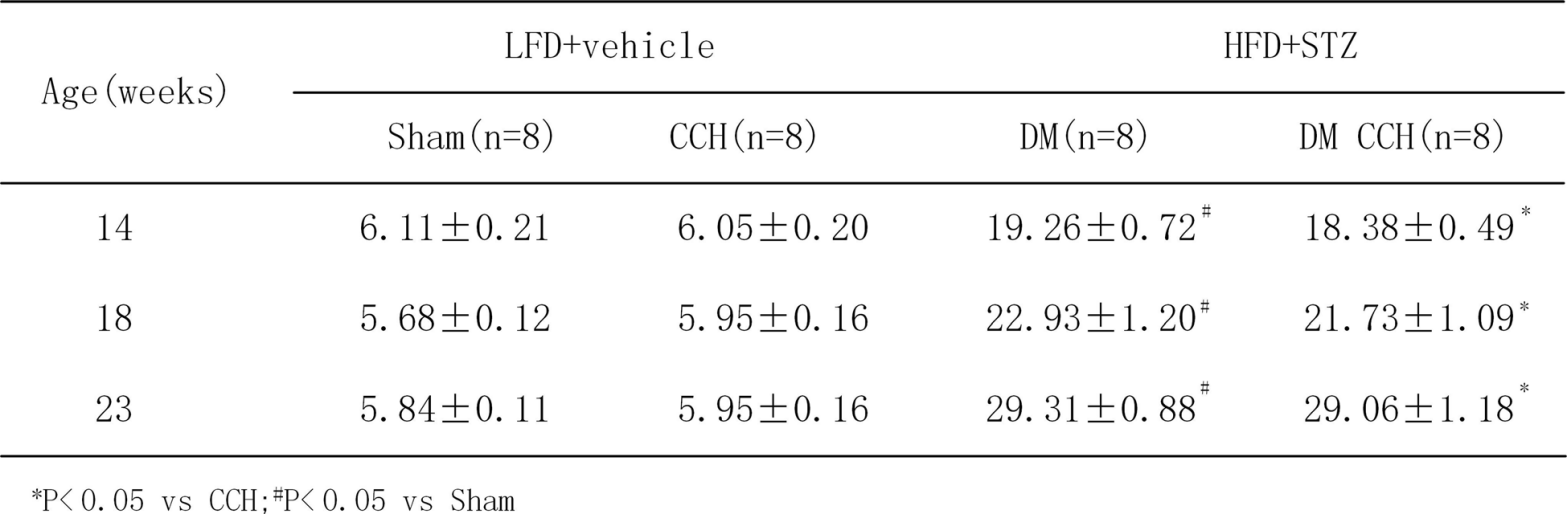
Body weight (g) of different groups rats

*P < 0.05 vs CCH;^#^*P* < 0.05 vs Sham

**Table 3:**
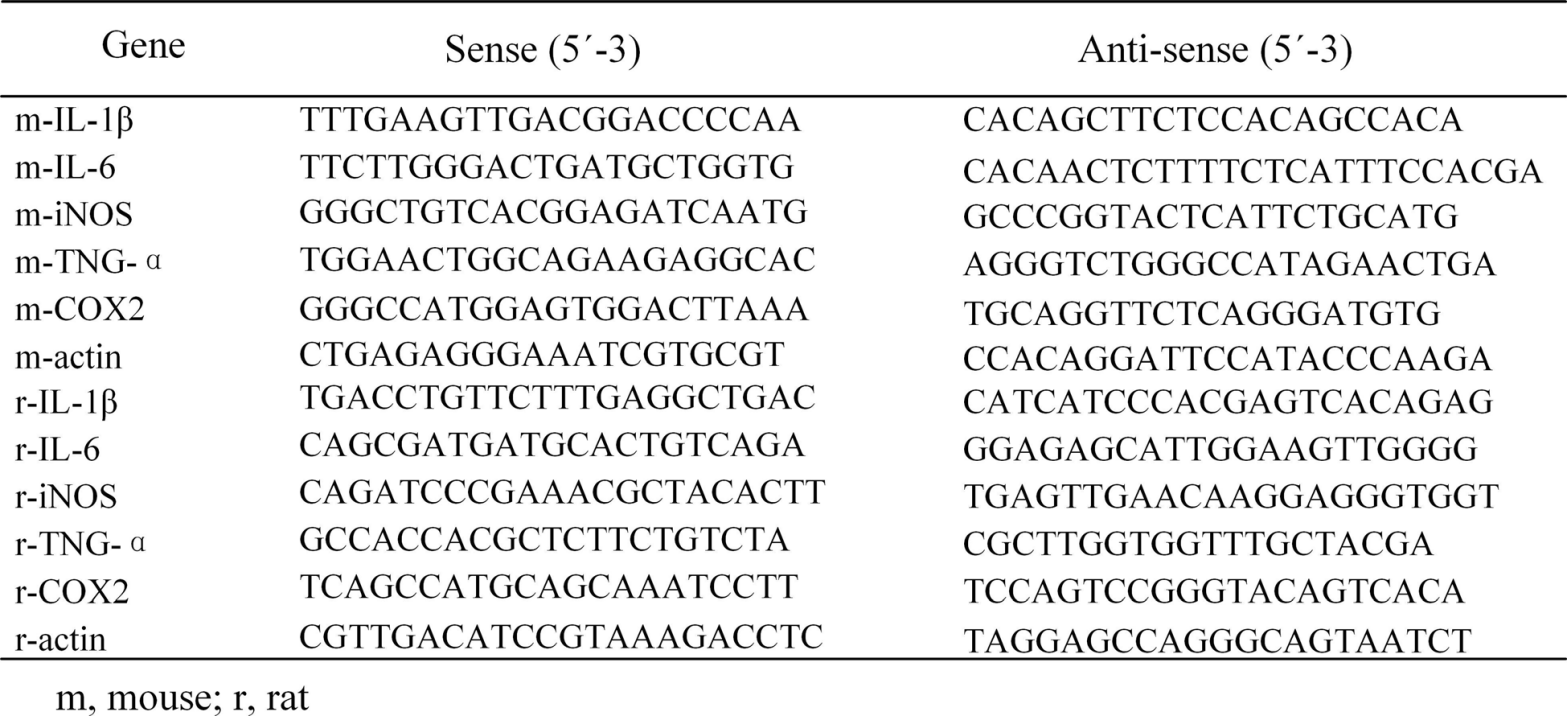
Fasting blood glucose (mmo1/L) of different groups of rats

*P < 0.05 vs CCH;^#^*P* < 0.05 vs Sham

In order to establish a simple and reliable model of high glucose and hypoxia in vitro, BV2 cells were treated with various concentrations of glucose (5.5, 11.1, 16.7, or 33 mM) under normal and hypoxic conditions (3% O_2_) for different times (0, 6, 12, 24, 48,72 h). A CCK-8 assay was used to evaluate the effect of both high glucose and hypoxia on cell viability. Data demonstrated that normal glucose (5.5 mM) and hypoxia (3% O_2_) ≤ 12 h resulted in little or no injury of BV2 cells, while hypoxia ≥ 24 h produced mild to moderate injury (Fig. 1f). Thus, 24 h of anaerobic incubation was a threshold, as cell survival rates decreased dramatically in response to in vitro hypoxia after this time point. Data also showed that cells exposed to different concentrations of glucose (5.5, 11.1, 16.7, or 33 mM) for 48 h alone could not elicit a decrease in cell viability. However, when cells were exposed to high glucose (33 mM) 24 h prior to a 24 h anaerobic incubation (3% O_2_), the cell viability was dramatically decreased as compared with hypoxia (3% O_2_) alone for 24 h (Fig. 1g). Thus, cells cultured under normoxic conditions for 24 h in medium supplied with high glucose (33 mM) followed by hypoxic conditions (3% O_2_) for 24 h were used as a model of DM and CCH in vitro in the following experiments.

### T2DM promoted CCH-induced learning and memory impairment

**Fig. 2:**
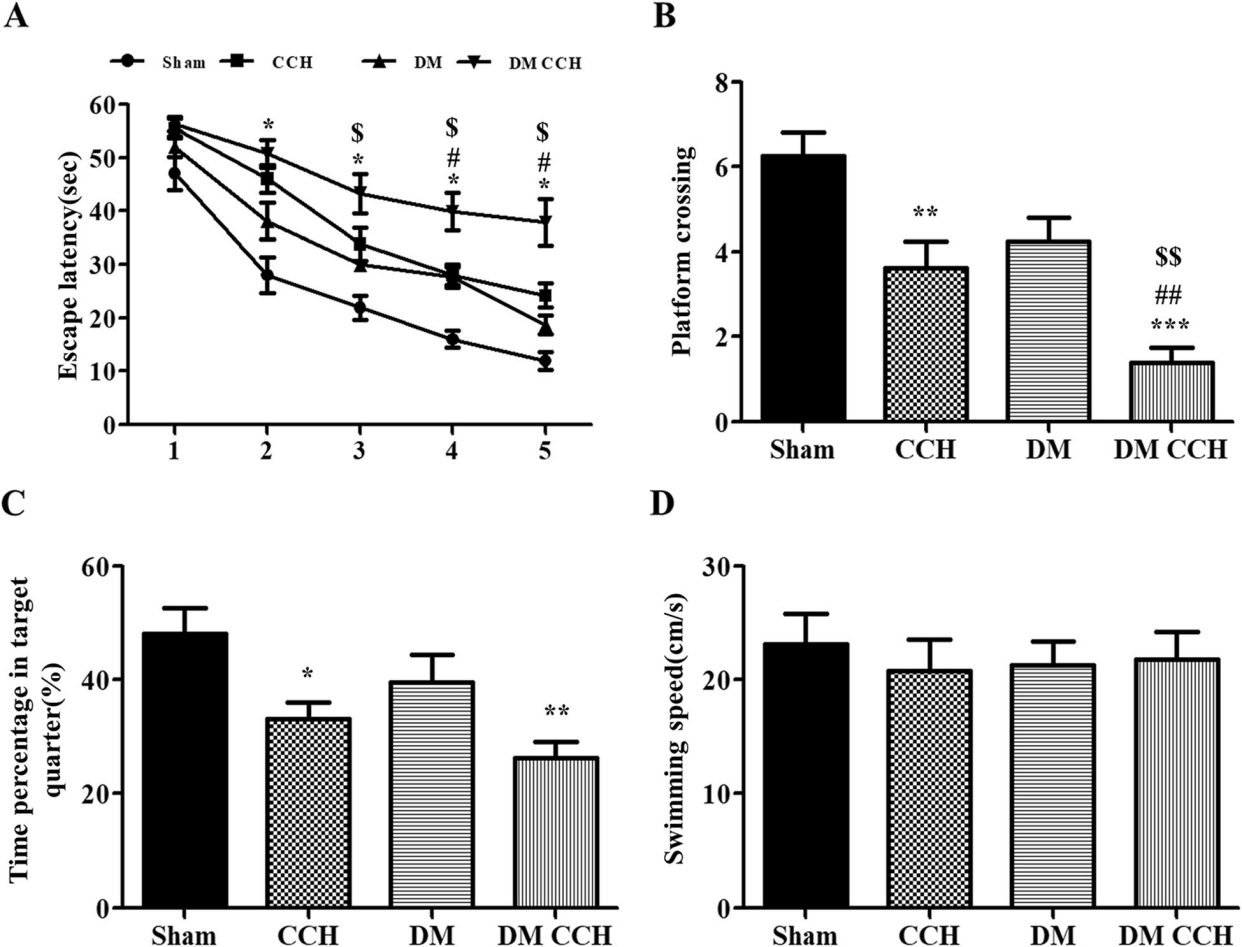
T2DM promoted CCH-induced learning and memory impairment. **a** The escape latency of rats in the training trials of the hidden platform task. **b** Frequency of platform crossing in the probe trial. **c** Percentage of time spent in the target quadrant in the probe trial. **d** Swimming speed in the probe trial. Values are expressed as mean ± SEM (n = 8). *P < 0.05; **P < 0.01 vs. Sham; ^#^*P* < 0.05; ^##^*P* < 0.01 DM CCH vs. CCH; ^$^*P* < 0.05; ^$$^*P* < 0.01 DM CCH vs. DM

Eight weeks after BCCAO or sham surgery, rats underwent MWM test to assess spatial learning and memory. There was no significant difference in escape latency on the first day among the four groups. Either DM or CCH alone slightly prolonged escape latency compared with the sham group. However, DM combined with CCH could significantly prolong escape latency relative to DM and CCH group, especially on days 4–5. But there was no significant difference in the DM group and CCH group in the whole test (Fig. 2a). In the probe trial, both the DM group and the CCH group performed poorer than the sham group, with fewer crossings over the original platform and less time spent in the target quadrant. Meanwhile, significant deterioration was observed in the DM CCH group compared with DM and CCH group (Fig. 1b, c). There was no difference among the experimental groups with respect to swimming speed (Fig. 1d). Thus, diabetes is synergistic with hypoperfusion to induce significant learning and memory impairment in our rat model.

### T2DM aggravated neuronal death in CCH-treated rats

**Fig. 3:**
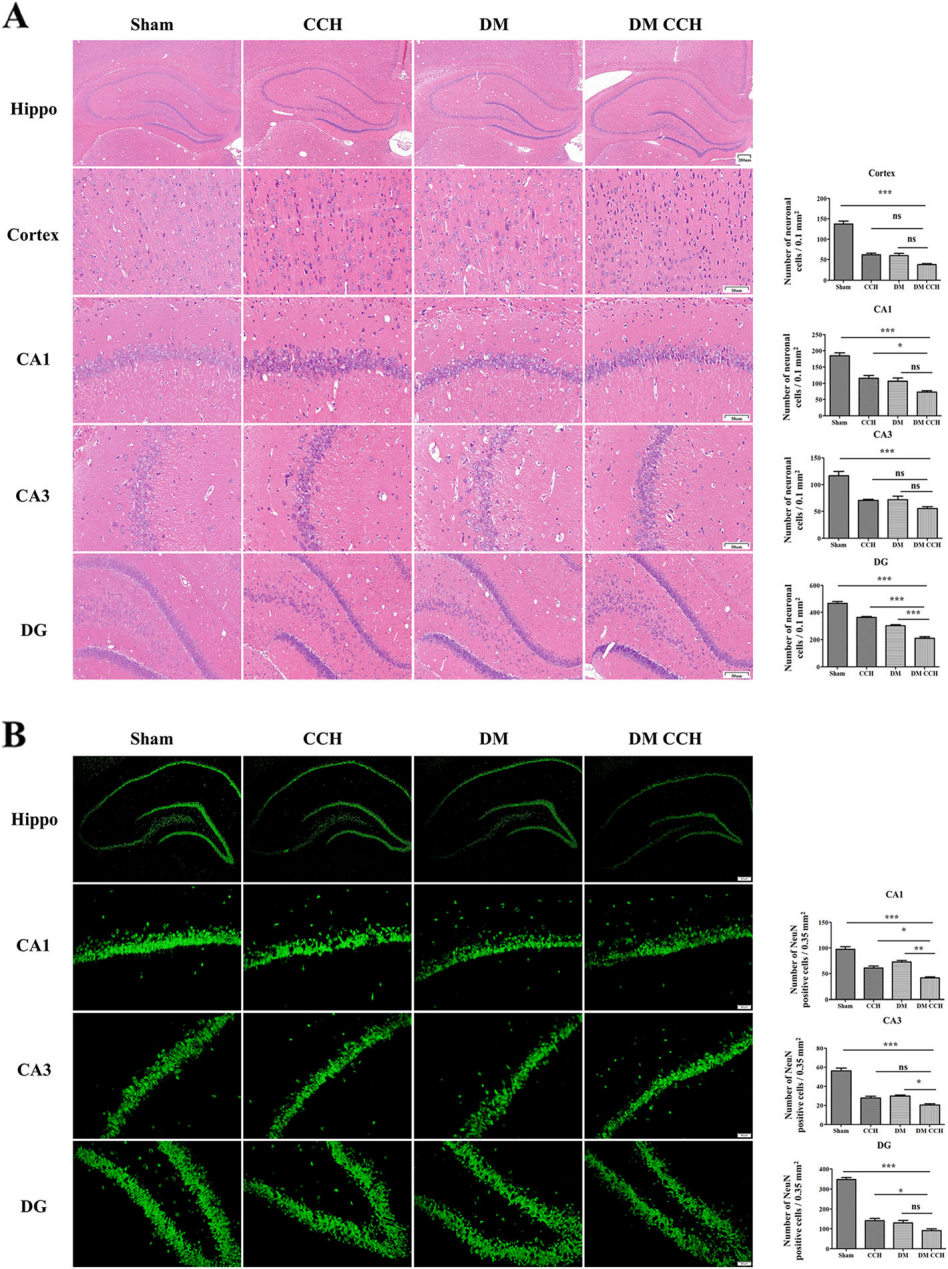
T2DM aggravated neuronal death in CCH-treated rats. **a**, **b** The sections of the hippocampus CA1, CA3, and DG region and cortex were obtained and stained by HE and NeuN (magnification × 40 or × 200, scale bar = 200 μm or 50 μm). Hippo, hippocampus

DM and CCH-induced neuronal death in the hippocampus and cortex was examined by HE staining. As shown in Fig. 3a, the neurons of the hippocampus and cortex in the sham group were intact, with round and full nuclei and clear boundary, while either DM or CCH group exhibited abnormal neuronal morphology with shrunken neurons and nuclear dark staining, which was more prominent in the DM CCH group. Furthermore, staining with NeuN, a neuron-specific marker, was performed to examine the density of neurons in the DG, CA1, and CA3 regions of the hippocampus. The DM or CCH group caused a decrease in the number of NeuN-positive cells in the CA1, CA3, and DG region of the hippocampus as compared with the sham group, and this decrease was larger in DM CCH group (Fig. 3b). These findings suggested that diabetes could aggravate neuronal death in CCH-treated rats.

### T2DM promoted microglia activation and pro-inflammatory cytokines production following CCH

**Fig. 4:**
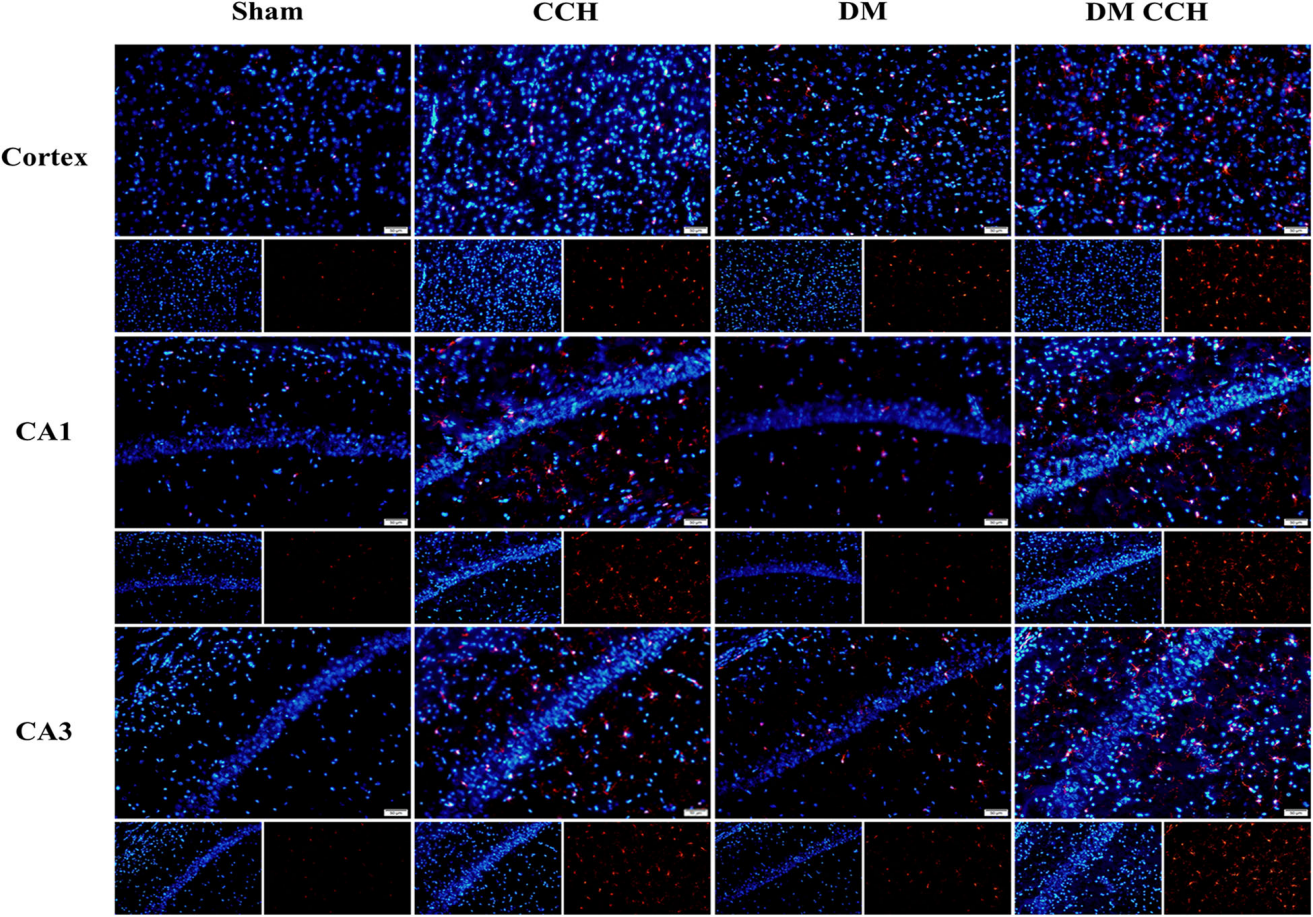
T2DM promoted microglia activation in CCH rats. Representative photomicrographs showed microglial activation at CA1, CA3, and cortex using an anti-ionized calcium-binding adaptor protein 1 (Iba1) antibody (red). Nuclei were stained with DAPI (blue). (magnification × 200, scale bar = 50 μm).

Glial activation is considered as a pathological hallmark in neurodegenerative disorders. Therefore, we investigated whether diabetes could promote microglia activation and subsequent production of neurotoxic pro-inflammatory cytokines in CCH-treated rats. At 8 weeks after surgery, brain sections were immunostained with anti-Iba-1 antibody to label the microglia. As shown in Fig. 4, activated microglia (Iba-1-stained cells) were rarely detected in the cortex and hippocampal CA1 and CA3 region in the sham group, while DM or CCH alone increased the number of Iba-1-positive microglial cells as compared with the sham group. However, Iba-1 staining was significantly higher in the DM CCH group compared with the remaining three groups.

**Fig. 5:**
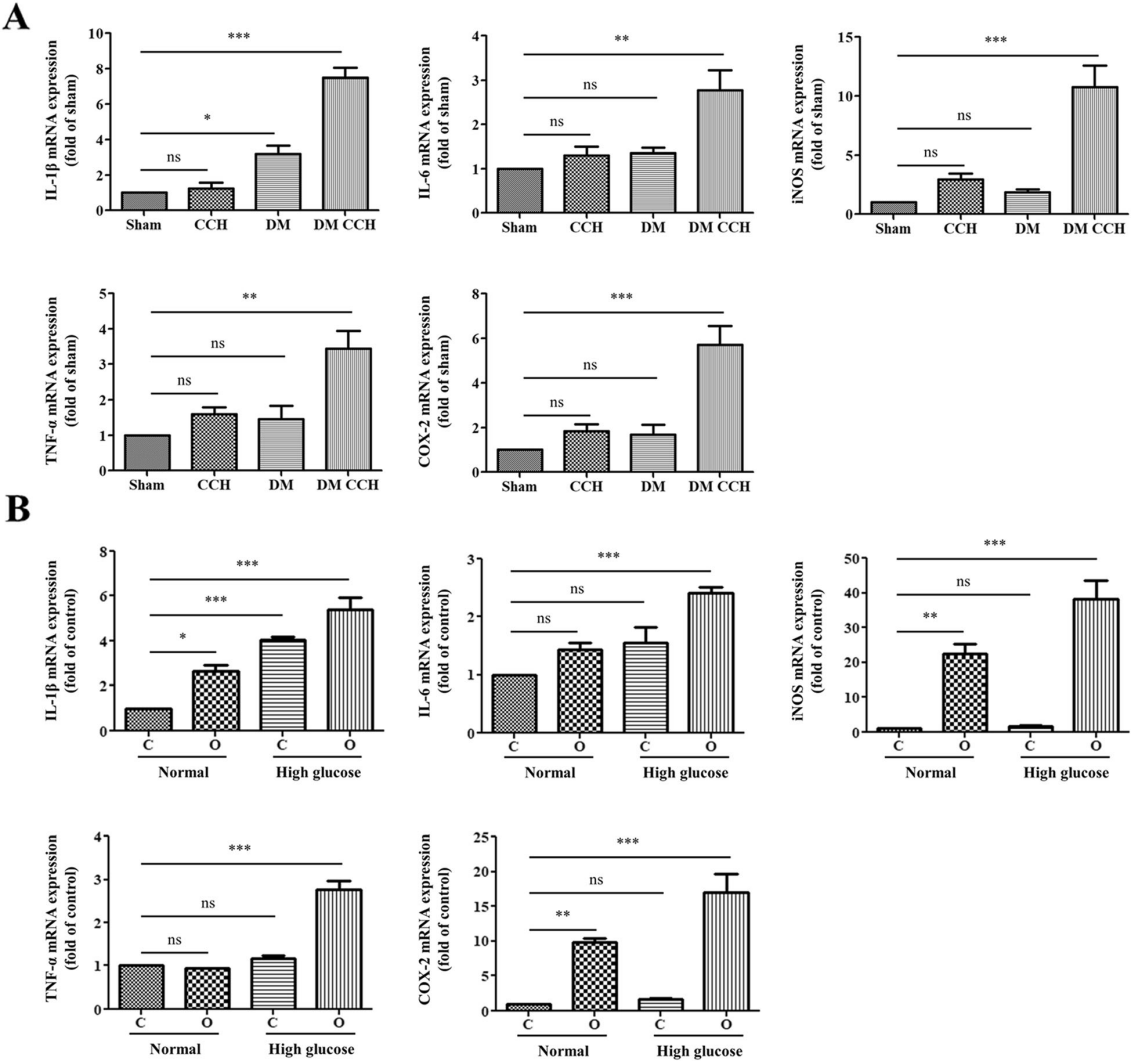
DM promoted pro-inflammatory cytokines production following CCH in vivo and in vitro. **a**, **b** The mRNA expression of pro-inflammatory cytokines (IL-1β, IL-6, TNF-α, iNOS, and COX2) was detected by RT-PCR. C, normoxia; O, hypoxia

We further detected the expression of pro-inflammatory cytokines, including IL-1β, IL-6, TNF-α, COX-2, and iNOS in each group by qRT-PCR. As shown in Fig. 5a, either DM or CCH group induced an increase of IL-1β, IL-6, TNF-α, COX-2, and iNOS levels as compared with the sham group, but without a statisticallysignificant difference**.** However, the combination of DM and CCH dramatically increased the expression of pro-inflammatory cytokines compared with the remaining three groups**.** Similarly**,** our in vitro model confirmed that hypoxia and high glucose could markedly accelerate the release of IL-1β, IL-6, TNF-α, COX-2, and iNOS compared with that of the control, hypoxia, or high glucose group (Fig. 5b). The results demonstrate that diabetes/high glucose promoted microglia activation and pro-inflammatory cytokine production following CCH/hypoxia.

### TREM-2-MAPK signaling was involved in T2DM-CCH rats and microglia under high glucose-hypoxia condition

**Fig. 6:**
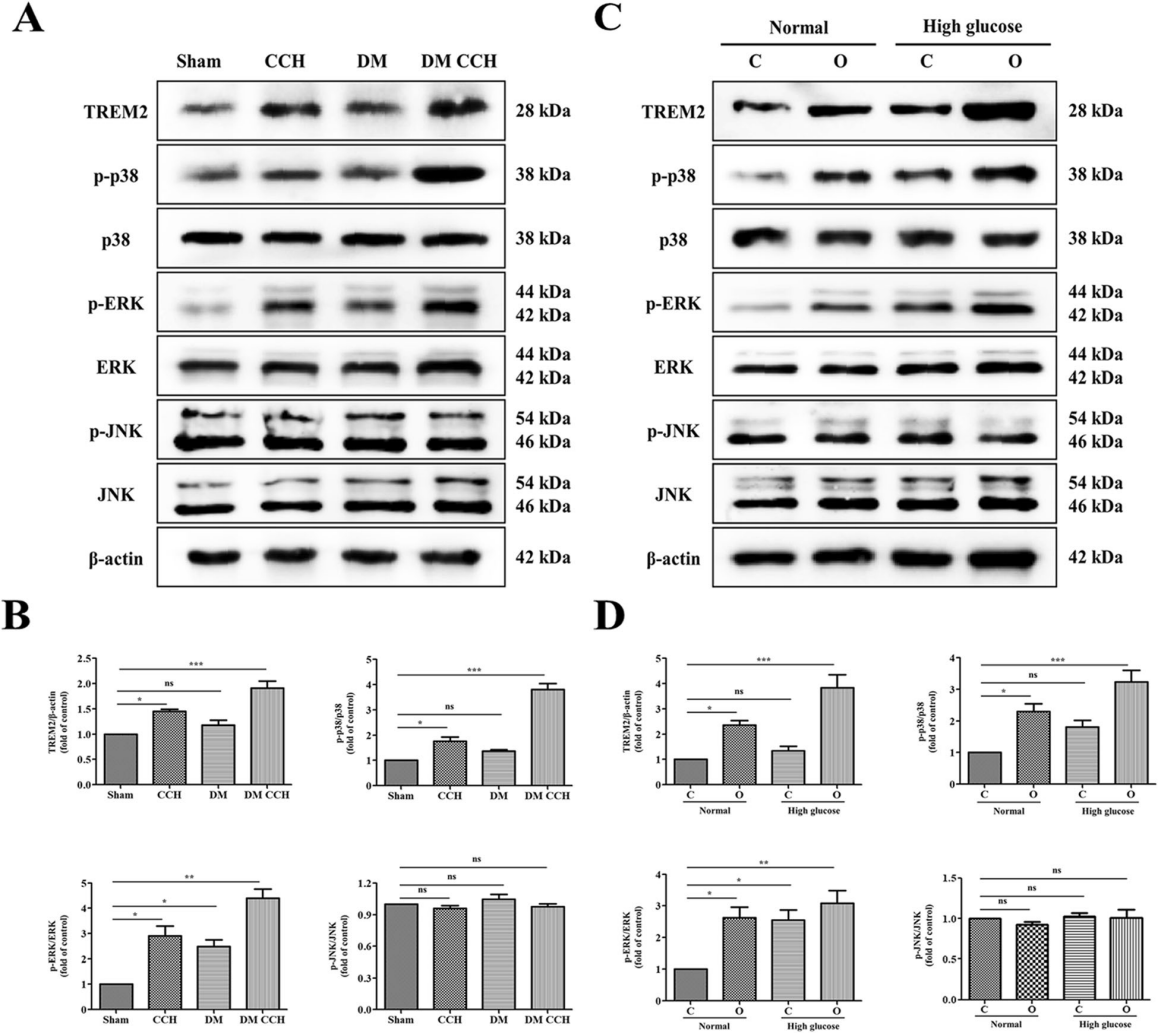
The TREM2 and MAPK signaling pathways were activated both in DM rats combined with CCH and microglial cells under high glucose and hypoxia. **a**, **c** Representative bands of Western blot data. **b**, **d** Quantitative analysis of the Western blot bands. *P < 0.05; **P < 0.01; ***P < 0.001 vs. Sham. C, normoxia; O, hypoxia.

It has been reported that both TREM-2 and MAPKs play an important role in signaling pathways mediating inflammation. Therefore, we first determined the levels of TREM-2 and MAPKs by Western blotting in vivo. As shown in Fig. 6 a and b, the expression of TREM-2, phosphorylated-ERK (p-ERK) and phosphorylated-p38 (p-p38) in either DM or CCH group was increased as compared with the sham group, but the increase was larger in the DM CCH group. No significant differences were found for p-JNK and the total levels of ERK, p38, and JNK. The results in vitro are similar to those in vivo. As shown in Fig. 6 c and d, the expression of p-ERK and p-p38 was also markedly increased in the hypoxia and high glucose group compared with that of the control, hypoxia, or high glucose group. Our data indicated that TREM-2-MAPK signaling might involve in regulating neuroinflammation in DM-CCH rats and microglia under high glucose-hypoxia condition.

### TREM-2 knockdown enhanced while TREM-2 overexpression inhibited pro-inflammatory cytokines in high glucose-hypoxia-stimulated microglia by targeting p38 MAPK

**Fig. 7:**
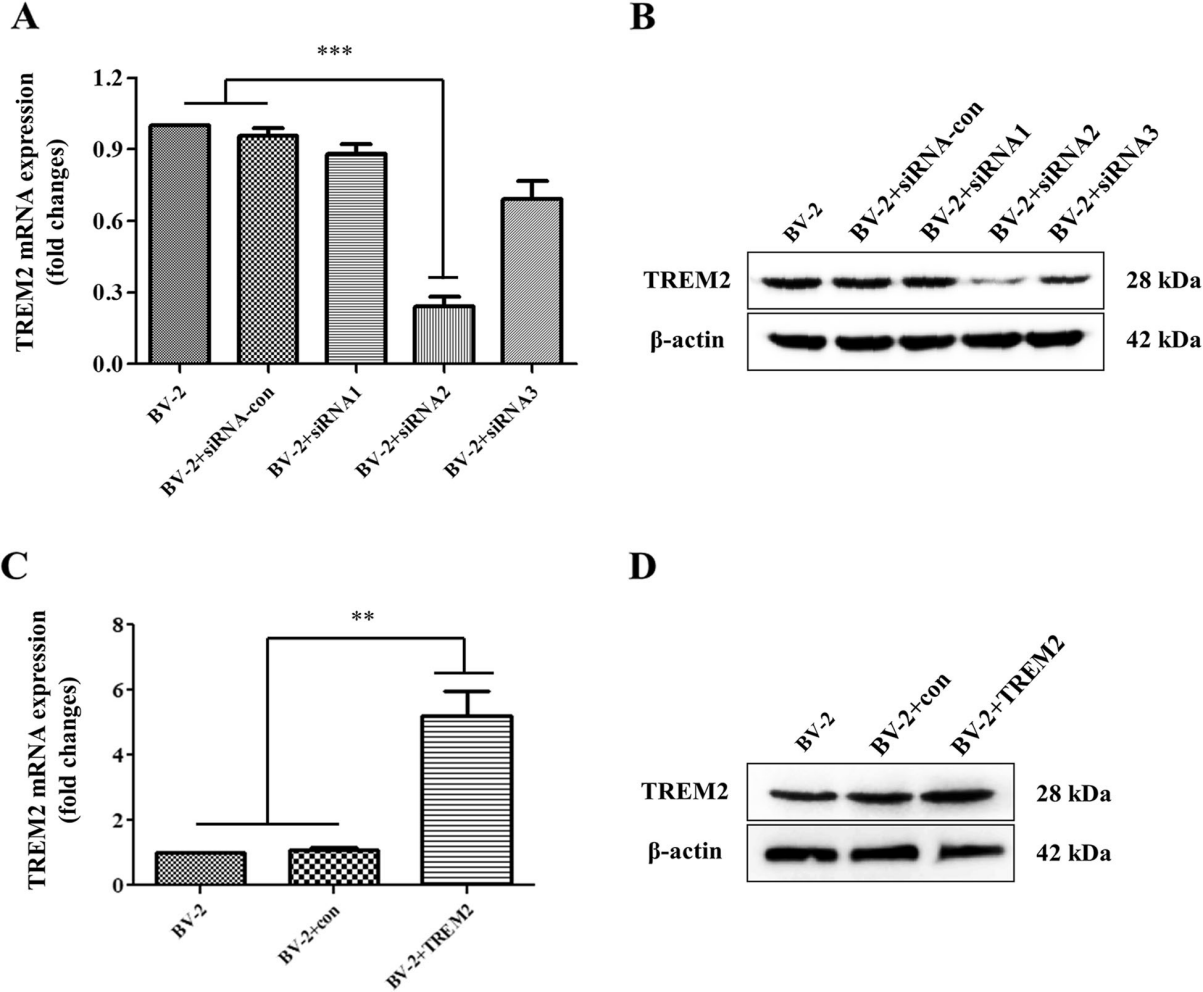
Characterization of TREM2 expression in different groups of BV2 cells. BV2 cells were transfected with TREM2 lentiviral particles or TREM2-specific siRNAs. The relative levels of TREM2 expression in different groups were determined by quantitative RT-PCR and Western blot. **a** Quantitative RT-PCR analysis of TREM2-specific siRNA mRNA transcripts. **b** Western blot analysis of TREM2 expression in BV2 cells transfected with TREM2-specific siRNAs. **c** Quantitative RT-PCR analysis of TREM2 mRNA transcripts. **d** Western blot analysis of TREM2 expression in BV2 cells transfected with TREM2 lentiviral particles. **P < 0.01; ***P < 0.001 vs. the BV2 cells

**Fig. 8:**
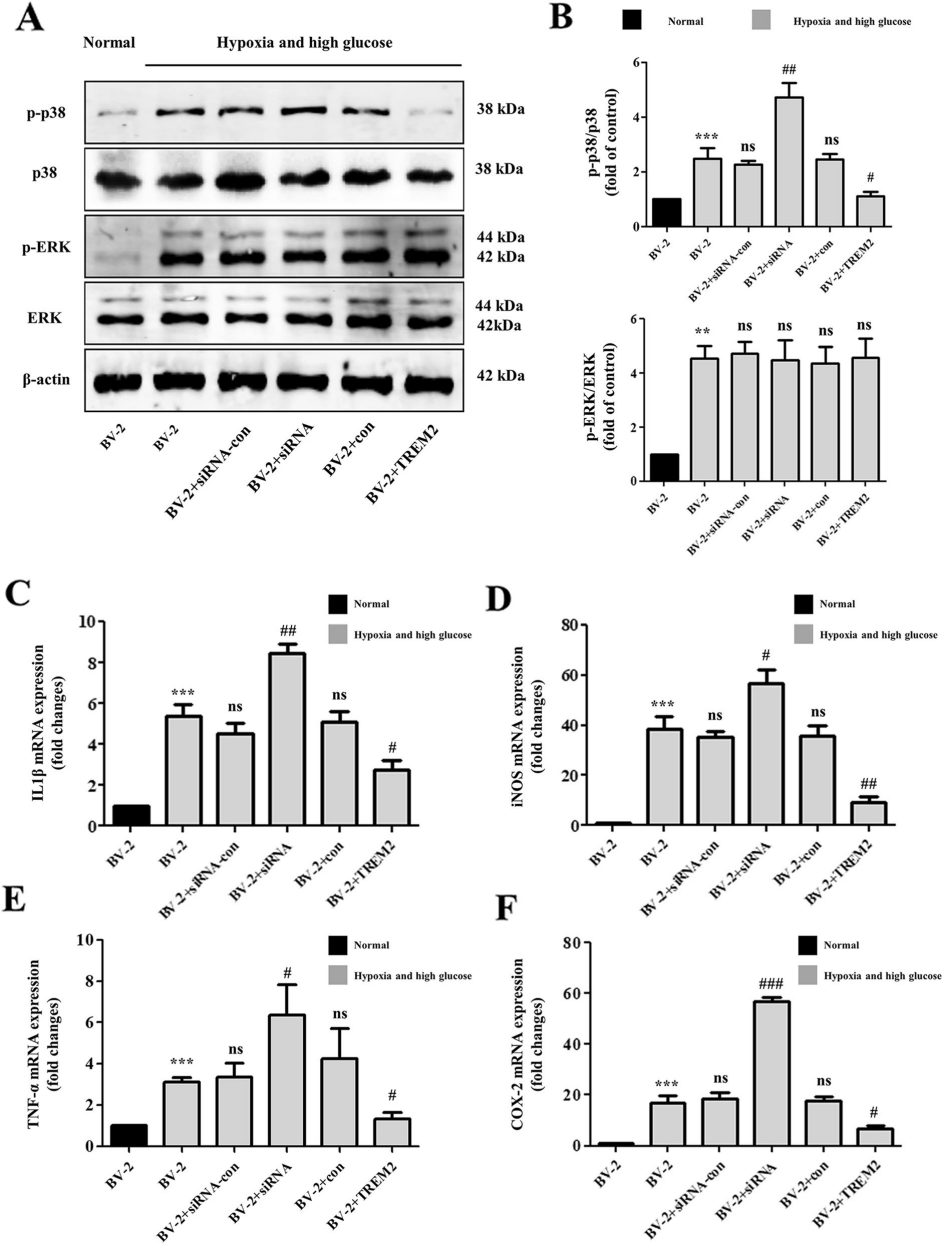
TREM-2 knockdown enhanced while TREM-2 overexpression inhibited pro-inflammatory cytokines in high glucose-hypoxia-stimulated microglia by targeting p38 MAPK. **a** Representative bands of Western blot data. **b** Quantitative analysis of the Western blot bands. **c**, **d** The mRNA expression of pro-inflammatory cytokines (IL-1β, TNF-α, iNOS, and COX2) was detected by RT-PCR. **P < 0.01; ***P < 0.001 vs. the BV2 cells (Normal); ^#^*P* < 0.05; ^##^*P* < 0.01; ^###^*P* < 0.001 vs. the BV2 cells (hypoxia and high glucose)

Recent studies of microglia under different conditions of neurodegeneration have revealed that TREM-2 is involved in mediating neuroprotective function and anti-inflammation. Surprisingly, however, our study found that TREM-2 was significantly elevated under hypoxia and high glucose conditions, which was contrary to our expectations. Therefore, We knocked out and overexpressed TREM-2 in vitro to further verify whether TREM-2 elevation is anti-inflammatory or is pro-inflammatory. At 48 h after transduced with the TREM-2-siRNA vector and TREM-2 lentiviral particles, the mRNA and protein levels of TREM-2 were significantly downregulated and upregulated, respectively (Fig. 7a–d). As shown in Fig. 8 c–f, TREM-2 knockdown substantially increased the expression of pro-inflammatory cytokines (IL-1β, TNF-α, COX-2, and iNOS) under hypoxia-high glucose condition, while TREM-2 overexpression induced a prominent reduction in the expression of pro-inflammatory cytokines (IL-1β, TNF-α, COX-2, and iNOS), nearly approaching the group under normal condition. To further explore whether TREM-2-mediated anti-inflammatory effects were specifically inhibiting the MAPK signaling pathway. Western blotting was carried out to study the expression of MAPK signaling molecules under hypoxia-high glucose condition. As shown in Fig. 8 a and b, TREM-2 overexpression inhibited and TREM-2 knockdown enhanced the phosphorylation of p38 without affecting the expression of phosphorylated-ERK and the total levels of ERK and p38. Taken together, the above results indicate that the anti-inflammatory effects exerted by TREM-2 are achieved through P38 MAPK signaling.

## Discussion

The present study investigated whether DM contributes to the development of CCH-induced cognitive dysfunction through the TREM-2-P38 MAPK signaling pathway and concomitant neuroinflammation. Upon BCCAO resulting in CCH resembling human VaD, we found that cognitive impairments were apparent in the HFD-STZ-induced DM rats. The exacerbating effects of DM on CCH outcomes were further confirmed in histopathological examination, in which neuronal death and microglia activation were increased with comorbid CCH and DM in rats. Similarly, both in vitro and in vivo results showed that hyperglycemia combined with CCH showed a more pronounced inflammatory response, which may be the result of the interaction between TREM-2 and MAPK signals. In vitro, knockdown and overexpression studies further demonstrated that TREM-2 can negatively regulate the p38 MAPK signaling under high glucose and hypoxia conditions. Overall, our findings suggest that TREM-2 negatively regulates p38 MAPK-mediated inflammatory response when DM was synergistically superimposed on CCH and highlight the importance of TREM-2 as a potential target of immune regulation in DM and CCH.

In order to generate a rat model that mimics the natural history of VaD patients comorbid with T2DM, a combination of HFD and low dose of STZ treatment, followed by BCCAO surgery was used to induce T2DM and CCH in vivo. T2DM is a complex metabolic disorder characterized by progressively declined insulin action (insulin resistance) and consequent inability to compensate for insulin resistance due to insufficient beta cell function [22]. As a major risk factor for T2DM, obesity is closely associated with insulin resistance, which can be induced by HFD [23, 24]. Moreover, a low dose of STZ has been widely used to induce T2DM in rodents by targeting beta cells [17, 25]. Accordingly, rats were fed a HFD to produce insulin resistance, followed by a low-dose STZ injection to induce beta cell dysfunction and subsequently hyperglycemia [26]. Our results showed a significant increase in body weight after 6 weeks of HFD, and blood glucose increased gradually over time after low-dose STZ injection. Subsequently, these rats exhibit clinically relevant characteristics of T2DM were further subjected to BCCAO surgery, which has been widely used to constitute the pathological condition of CCH. Our study found that CBF decreased significantly by more than 70% immediately after BCCAO, comparable with that observed in clinical patients [18]. Additionally, we found that compared with the CCH group, CBF decrease was more pronounced in the DM-CCH group, but without statistical difference. Collectively, the present study indicates that the HFD-STZ-treated and BCCAO rat can serve as an alternative animal model for CCH in T2DM in vivo simulating the human syndrome. The duration of hypoxia and glucose concentrations in vitro systems should be of practical relevance to the brain in vivo. However, the duration of hypoxia and glucose concentrations to induce hypoxia and hyperglycemia was varied in different in vitro models [20, 21, 27]. Our data showed that cells exposed to high glucose (33 mM) 24 h prior to a 24 h anaerobic incubation (3% O_2_) resulted in moderate microglial cell death as compared with hypoxia (3% O_2_) alone for 24 h. The adverse effects of hypoxia and high glucose are agreement with previous reports [28, 29]. We thus determined that high glucose (33 mM) 24 h prior to a 24 h anaerobic incubation (3% O_2_) mimicked hypoxia and hyperglycemia in subsequent experiments.

BCCAO produces a chronic, global hypoperfusion state, which proved to be a classical animal model for the study of VaD and CCH. Neurons, especially in the hippocampus, are closely related to spatial learning and memory functions, and are susceptible to ischaemic conditions. Delayed degeneration of these neurons caused by global ischemia results in cognitive deficits. Neuronal degeneration and memory deficits caused by CCH have been extensively reported [7, 30]. Consistently, we have previously found that neuronal injury and cognitive impairment are prominent at 8 weeks following BCCAO [18]. Similarly, studies also found that STZ-induced DM could aggravate neuronal apoptosis and cognitive dysfunction in rodents [31]. In our present Morris water maze task, spatial learning and memory impairment were more pronounced in the DM-CCH group than those in the CCH group at 8 weeks post-surgery, as indicated by an increase in escape latency, fewer crossings over the original platform and less time spent in the target quadrant. Moreover, we found that DM promoted CCH-induced neural damage, as indicated by decreased positive cells in NeuN staining and shrunken neurons and dark stained nuclei in HE staining. These results suggest that DM could aggravate CCH-induced spatial learning and memory impairments and neuronal injury in rats.

It is well established that inflammatory responses, including activation of glial cells and production of inflammatory cytokines such as IL-1β, IL-6, COX-2, iNOS, and TNF-α, induced by CCH could further result in neuronal cell death and cognitive deficits [32, 33]. Diabetes was also shown to increase microglial activation and the production of pro-inflammatory cytokines in previous studies [34, 35]. Collectively, neuroinflammation might play a crucial role in the initiation and progression of DM combined with CCH. Consistent with these previous findings, we observed that DM could efficiently promote CCH-induced microglial activation and inflammatory responses. In vitro results also indicated that high glucose synergistically promoted hypoxia-induced release of pro-inflammatory factors. CCH causes a cascade of pathological processes, during which diverse signaling pathways are activated, including TREM-2 and MAPKs [36, 37]. The phosphorylation of MAPK-related signaling molecules, such as ERK, JNK, and p38 MAPK, have been reported to induce the release of many pro-inflammatory mediators such as IL-1β, IL-6, COX-2, iNOS, and TNF-α, in microglia [38]. As an innate immune receptor predominately expressed in microglial cells within the CNS, TREM-2 has been shown to mediate the primary function of microglia, such as suppression of pro-inflammatory cytokines and promotion of phagocytosis. It has been reported that TREM-2 could inhibit neuroinflammation by negatively regulating the MAPK signaling pathways in experimental models of Parkinson's disease [39]. Our in vivo study found that DM could further activate the P38 and ERK signaling pathways rather than the JNK signaling pathway in CCH rats, and unexpectedly also increased the expression of TREM-2, which was expected to be decreased. Our in vitro high glucose-hypoxia experiment further confirmed these results. Although most studies show that TREM-2 has an anti-inflammatory effect, some studies also found that TREM-2 has pro-inflammatory properties, possibly related to the disease model [40–42]. To explore whether TREM-2 elevation is anti-inflammatory or is pro-inflammatory, TREM-2 was knocked out and overexpressed in vitro using siRNA and lentiviral technique, respectively. Results showed that the decrease in TREM-2 expression further augmented the activation of P38 MAPK signaling instead of ERK signaling, leading to detrimental exaggeration of neuroinflammation, while TREM-2 overexpression inhibited the expression of P38 MAPK rather than ERK, resulting in neuroinflammatory remission.

## Conclusions

Taken together, our studies suggest that DM can synergistically promote cognitive dysfunction induced by CCH through p38 MAPK-mediated neuroinflammation and consequently increased neuronal cell death in the hippocampus and cortex. Additionally, the negative regulatory effect of TREM-2 on inflammation might be insufficient to antagonize DM-CCH-induced hyperactive p38 MAPK. These findings indicate that TREM-2 may serve as a potential therapeutic target for vascular dementia combined with DM. However, further studies are needed to explore the role of TREM-2 in regulating p38 MAPK signaling pathways in vivo models.

## Data Availability

All data generated or analyzed during this work are included in this article.

## Abbreviations

DM: Diabetes mellitus
HFD: High-fat diet
LFD: Low-fat control diet
VaD: Vascular dementia
CCH: chronic cerebral hypoperfusion
BCCAO: Bilateral common carotid artery occlusion
CBF: Cerebral blood flow
LSCI: Laser speckle contrast imaging
TREM2: Triggering receptor expressed on myeloid cells 2
MAPK: Mitogen-activated protein kinases
STZ: Streptozotocin
